# Protective effect of melatonin against Adriamycin-induced cardiotoxicity

**DOI:** 10.3892/etm.2013.989

**Published:** 2013-03-06

**Authors:** YAN ZHANG, LIXIN LI, CHENG XIANG, ZHIQIAN MA, TIAN MA, SHUCHAI ZHU

**Affiliations:** 1 Departments of Oncology, The First Hospital of Shijiazhuang, Shijiazhuang, Hebei 050010; 2 Clinical Laboratory, The First Hospital of Shijiazhuang, Shijiazhuang, Hebei 050010; 3 Pharmacy, The First Hospital of Shijiazhuang, Shijiazhuang, Hebei 050010;; 4 Department of Radiotherapy, The Fourth Hospital of Hebei Medical University, Shijiazhuang, Hebei 050011, P.R. China

**Keywords:** melatonin, Adriamycin, cardiotoxicity, breast cancer

## Abstract

The aim of this *in vivo* study was to explore the protective properties of melatonin against Adriamycin-induced myocardial toxicity. A rat model of breast cancer was established and the rats were randomly divided into the blank group (Blank), the solvent group [Diss; dehydrated alcohol: physiological saline (1:9)], the Adriamycin group (ADM), the melatonin group (MLT) and the melatonin + Adriamycin group (M+A). The concentrations of lipid peroxide (LPO), superoxide dismutase (SOD) and glutathione peroxidase (GSH-Px) in myocardial tissues were detected, the changes in myocardial tissues were observed using light microscopy and electron microscopy, and the 1-month survival rates of each group of rats were compared. Breast cancer was established in 116 rats. In the ADM group, the concentration of LPO was higher and the concentrations of SOD and GSH-Px were significantly lower than those in the blank group. In the M+A group, compared with the ADM group, the concentration of LPO was lower (P<0.05) and the concentrations of SOD and GSH-Px were higher (P<0.05). It was observed using light and electron microscopy that the myocardial injuries to the M+A group were significantly alleviated in comparison with those in the ADM group; the 1-month survival rate in the M+A group was higher than that in the ADM group. Melatonin may have a protective role in the myocardium by reducing Adriamycin-induced myocardial oxidative damage.

## Introduction

Breast cancer is a malignant tumor with a high incidence in females from more developed western countries. Although historically the incidence of breast cancer in women in China was low, it has been on the increase in the past 20 years. In large cities, such as Beijing and Shanghai, breast cancer is the most common type of malignant tumor diagnosed in women. As the country with the highest population in the world, there are a large total number of cases annually. Anthracycline chemotherapeutic agents, including Adriamycin, are among the main agents used for breast cancer chemotherapy. Adriamycin has been essential in breast cancer therapy, particularly in prolonging the survival of patients with advanced and metastatic breast cancer. However, due to the dose-limiting cardiotoxicity of Adriamycin, it is not suitable for use in patients with cardiac disorders. The administration of Adriamycin may cause varying degrees of myocardial toxicity, seriously affecting the patients’ quality of life, and in certain cases has caused toxicity-related mortality (1–3).

In recent years, studies of myocardial toxicity have been reported, but no drug has been satisfactorily applied in a clinical environment (4–6). With developments in the treatment of malignant tumors, tumor reduction and disease relief in the short term are no longer sufficient and steps to improve quality of life and prolong the survival of patients are drawing an increasing amount of attention. There has been a strong clinical demand for cardioprotective drugs for use in tumor patients (7). There is a need for a drug that is not only helpful in the treatment of breast cancer, but also alleviates Adriamycin-induced cardiotoxicity.

Melatonin is an endocrine hormone naturally present in the human body, which has a complicated and important physiological role. Studies have confirmed that melatonin has an inhibitory effect on multiple malignant tumors; the antitumor effects in patients with ER^+^ breast cancer have been reported (8–11). The inhibitory effect on breast cancer cells was confirmed again at the cellular level in our previous research (12,13). Notably, the resistance reversal effect and sensitizing mechanism of Adriamycin have been observed. Additionally, melatonin, currently the strongest known antioxidant (14–17), has been confirmed to have a significant multi-organ protective effect via a complex mechanism (18–22).

It would be of interest to determine whether melatonin has a cardioprotective effect that would aid the anticarcinogenic effect of Adriamycin and, if so, to understand the mechanism. Melatonin, therefore, may be an excellent adjuvant drug in breast cancer chemotherapy, which would be worthy of further study. A rat model of ER^+^ breast cancer was established in order to investigate melatonin’s effects. The condition of the human body was simulated as closely as possible in order to observe the cardiotoxicity and the quality of life in rats concurrently treated with melatonin and Adriamycin.

## Materials and methods

### Animals

In this study, 140 healthy female Sprague-Dawley (SD) rats, weighing ∼200–250 g, were purchased from the Animal Center of Hebei Medical University. The rats were maintained in light-dark (LD) conditions. LD involved 12 h of light (from 6:00 to 18:00) and 12 h of darkness (from 18:00 to 6:00). The rats had free access to water and food, and were maintained in a room with a temperature of 25±2°C and humidity of 45–50%. The rats entered the trial after 3 weeks of synchronization. This study was carried out in strict accordance with the recommendations in the Guide for the Care and Use of Laboratory Animals of the National Institutes of Health. The protocol for animal use was reviewed and approved by the Institutional Animal Care and Use Committee (IACUC) of the First Hospital of Shijiazhuang, Hebei.

### Establishment of the rat model of breast cancer

According to the Russo method (23), of the 140 female SD rats, 130 were injected with N-nitroso-N-methylurea (MNU; Sigma-Aldrich, St Louis, MO, USA) to induce and establish the breast cancer models, and the remaining 10 rats underwent an intraperitoneal injection with a solvent which is used to dissolve MNU.

According to the Dagar method (24), the rats were weighed and numbered. The rats were intraperitoneally injected with a 50 mg/kg dose of MNU. After being weighed 2 weeks later, they were injected again with the same dosage of MNU. The tumor growth in the rat breast was observed on a weekly basis for the timely evaluation of successful cases.

### Animal groups

The rats were divided into the blank control group (Blank); the solvent control group [Diss; dehydrated alcohol: physiological saline (1:9)]; the melatonin group (MLT) who were injected with a 10 mg/kg dose of melatonin (Sigma-Aldrich) once a day for a total of 15 times; the Adriamycin group (ADM) who were injected with a 2.5 mg/ml dose of Adriamycin every other day for a total of 7 times; and the melatonin + Adriamycin (M+A) group. From the first day in the M+A group the rats were treated with a 10 mg/kg dose of MLT once a day for 15 days, and from the third day the rats were also treated with a 2.5 mg/ml dose of ADM every other day. MLT was injected intraperitoneally prior to ADM. On the 18th day, a number of each group of rats were sacrificed and taken for analysis; the remaining rats continued to be observed to ascertain the survival rates.

### Detection of oxidative indices

Each group of rats underwent an intraperitoneal injection of 30 mg/kg pentobarbital sodium as an anesthetic, prior to opening the chest and the quick removal of the heart, which was flushed with physiological saline three times and dried with filter paper. After being weighed, the heart was prepared as a 10% homogenate with a 0.2 M/l phosphate buffer solution (pH 8.6) in an ice water bath to determine the concentration of lipid peroxide (LPO) and the activities of superoxide dismutase (SOD) and glutathione peroxidase (GSH-Px) in the myocardial tissue.

### Preparation of myocardial tissue samples for light microscopy

The rats underwent anesthesia as described above, followed by the removal of the heart. The interventricular septum was dissected and the myocardial tissues were removed from the left ventricle. The myocardial tissues were cut into 8–10 blocks of 1 mm^3^ along with the striation of muscle fiber and fixed in 10% paraformaldehyde phosphate buffer solution for pathological sectioning and hematoxylin and eosin (H&E) staining. The tissues were observed and photographic images captured under a light microscope using low and high magnification.

### Preparation of samples for electron microscopy

The steps for processing the myocardial tissue samples for electron microscopy included: sampling, pre-fixation, washing, post-fixation, washing, dehydration, saturation, embedding, polymerization, ultrathin sectioning, observation and photographic image capture.

### Statistical analysis

The data were analyzed with the statistical software SPSS 13.0 (SPSS, Inc., Chicago, IL, USA). The oxidative indices were compared using t-tests and the 1-month survival rate was compared with the Chi-square test. P<0.05 was considered to indicate a statistically significant difference

## Results

### Tumors and groups

In total, 140 female SD rats were used in this study. Of the 10 rats in the MNU Diss group, there appeared to be one death without an identifiable cause and the other rats were in a good condition, without the generation of any tumors. Among the 130 rats who underwent MNU injection, four rats died during the injection and three rats who had generated tumors died before they were placed into groups. In total, seven rats did not generate tumors and 116 rats did generate tumors and were randomly placed into groups. The tumor initiation rate was 91.5% (119/130). In each group, eight rats were randomly selected and dissected to remove the tumors and hearts and then sacrificed, and the remaining rats (24 rats in the blank group, 24 in the solvent group, 22 in the MLT group, 24 in the ADM group, 22 in the M+A group) were observed for six months in order to determine mortality rates.

### Oxidative indices

The oxidative indices included LPO, SOD and GSH-Px concentrations in the myocardial tissues. There were no marked differences in each index among the Blank, Diss and MLT groups. The concentration of LPO in the myocardial tissues in the ADM group was significantly higher when compared with that in the M+A group (P<0.05). The concentrations of SOD and GSH-Px in the ADM group were significantly lower when compared with those in the M+A group (P<0.05, Table I).

### General characteristics of the hearts

The shapes, sizes and weight of the hearts in the MLT, Blank and Diss groups were close to normal. The hearts of the rats in the ADM group appeared to be markedly congested and swollen, with increased volumes and visible petechiae on the pericardium. The hearts in the M+A group were essentially normal in shape, with mild congestion, but without visible petechiae on the pericardium.

### Appearance of hearts under light and electron microscopy

There were no marked anomalies in the Blank, Diss and MLT groups, either under a light microscope with low magnification or under an electron microscope for H&E-stained myocardial sections.

**Figure 1:**
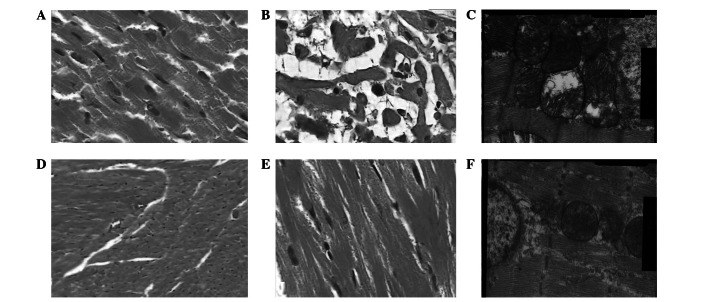
(A) Adriamycin-induced myocardial injuries and muscle bundle fractures were visible under a light microscope with low magnification in the ADM group (magnification, ×100); (B) Adriamycin-induced myocardial mucinous degeneration was visible under a light microscope with high magnification in the ADM group (magnification, ×400); (C) Adriamycin-induced organelle injuries in myocardial cells and serious mitochondrial cavitation were visible under a light microscope with high magnification in the ADM group (magnification, ×20.0KX); (D) Essentially normal cardiac muscles were visible under a light microscope with low magnification in the M+A group (magnification, ×100); (E) Only mild granular changes were visible under a light microscope with high magnification in the M+A group (magnification, 400×); (F) Adriamycin-induced organelle injuries in myocardial cells and serious mitochondrial cavitation were visible under an electron microscope with high magnification in the M+A group (magnification, ×20,000). Blank, no intervention; Diss, solvent intervention; MLT, melatonin intervention; ADM, Adriamycin intervention; M+A, melatonin + Adriamycin intervention.

In the ADM group, under a light microscope with a low magnification, a large number of cardiac muscle bundle fractures were observed, with mucus visible between the muscle bundles (Fig. 1A). Under a light microscope with a high magnification, it was observed that the cardiac muscles appeared to be disordered with severe mucinous degeneration (Fig. 1B). Under an electron microscope, it was observed that the matrix in the nuclear side appeared to have a marked edema, where the majority of the ridges and a few of the mitochondrial membranes appeared to be fused, indistinct or missing, some of the chromosomes appeared to have undergone the cavitation phenomenon, glycogen granules were significantly reduced under an electron microscope and parts of the coxae were either arranged in an unorganized manner or missing (Fig. 1C).

Under a light microscope with a low magnification, it was observed that the cardiac muscle bundles were essentially normal in the M+A group, but mild bundle fractures were present in a few areas (Fig. 1D). Mild granular degeneration was observed under a light microscope with high magnification (Fig. 1E). Under an electron microscope it was observed that a few sections of the mitochondrial ridges appeared to be fused and indistinct and the glycogen granules were reduced, but no cavitation phenomenon was observed in the chromosomes (Fig. 1F).

### Survival rates after 1-month

The 1-month survival rates of the remaining rats in each group were analyzed and are shown in Table II. The 1-month survival rates were 4/16 in the Blank group and 6/16 in the Diss group, without a statistically significant difference (P=0.35), 14/14 in the MLT group, with a total survival rate significantly higher than in the previous two groups (P=0.000), 5/16 in the ADM group and 11/14 in the M+A group, with the latter higher than the former (P=0.012), without a statistically significant difference between the MLT group and the M+A group (P=0.11).

## Discussion

Melatonin is an endocrine substance naturally present *in vivo* that is secreted by the pinealocyte. It is widely distributed in various organs of the human body and plays an important and complicated biological function *in vivo*. Studies have confirmed that melatonin has a multi-organ protective effect, which indicates that melatonin may also play a role in myocardial protection by promoting the anti-tumor effect of Adriamycin (21,25–28). In this study, rat models of ER^+^ breast cancer were established in order to compare the cardiotoxicity in the ADM group, with exposure to Adriamycin, and the M+A group, with concurrent exposure to melatonin and Adriamycin. It was observed in the general samples that the volumes of the hearts of the rats in the ADM group were larger, with marked congestion and swelling, and varying severities of petechiae on the pericardium. By contrast, the hearts in the M+A group were almost normal in shape and color, with visible congestion in severe cases, but without visible petechiae on the pericardium. Under a light microscope with a low magnification, it was observed that the myocardial tissues of the ADM rats appeared to have a large number of cardiac muscle bundle fractures, with mucus between the cardiac muscles (Fig. 1A). It was observed, under a light microscope with high magnification, that the cardiac muscles were arranged in a disorderly manner and appeared to have severe mucinous degeneration (Fig. 1B). It was also observed, under a light microscope with low magnification, that the cardiac muscle bundles were essentially normal in the M+T group, with no marked bundle fractures (Fig. 1D); mild granular degeneration was visible under a light microscope with high magnification (Fig. 1E). Thereafter, changes in organelle levels were observed using electron microscopy and it was demonstrated that the organelles in the myocardial cells were badly damaged in the ADM group and the majority of the mitochondria appeared to undergo the cavitation phenomenon (Fig. 1C). There were only mild injuries to the mitochondria in the M+A group (Fig. 1F). From different levels of morphology, it was demonstrated that the myocardial injuries in the M+A group, with intervention of melatonin, were alleviated compared with those in the ADM group with a single application of Adriamycin, which was consistent with our expectations, indicating that melatonin has a protective effect against Adriamycin-induced myocardial toxicity.

Some hypotheses concerning the mechanism of anthracycline-induced myocardial injuries are as follows: i) the oxidative stress effects produce a large number of free radicals, causing myocardial injuries via oxidation; ii) calcium overload and energy metabolism disorder may cause lipid peroxidation in myocardial cells; iii) the tyrosine residing in myocardial cells became nitrated by Adriamycin; iv) the myocardial cells appeared to undergo direct apoptosis. Free radical damage and lipid peroxidation in myocardial cells represented significant anthracycline-induced myocardial injuries. Therefore, the scavenging of free radicals is an important measure for preventing myocardial injuries (29).

Superoxide dismutase (SOD) scavenges free radicals *in vivo*. It catalyzes the conversion of superoxide radicals into hydrogen peroxide and oxygen molecules and is key in resisting cell damage caused by oxygen free radicals. Glutathione peroxidase (GSH-Px) is an important peroxide-decomposing enzyme widely found *in vivo* and is a detoxification enzyme that scavenges hydrogen peroxide and other organic peroxides. It protects the structure and the function of the cell membrane from peroxide interference and damage through action as an antioxidant. LPO is produced by the polyunsaturated fatty acids in the cell membrane structure *in vivo* with the influence of oxygen free radicals. The peroxidation of lipid membranes may damage the membrane structure and cell function, causing a variety of diseases. Floyd *et al*(30) suggested that SOD, GSH-Px and LPO may be important indicators in pharmacodynamic studies of Adriamycin-induced cardiotoxicity with drug intervention, as they are closely related to the degree of myocardial injury. In the current study, SOD and GSH-Px were observed to be negatively correlated with Adriamycin-induced myocardial injuries, while LPO was positively correlated (Table I), indicating that these three indicators may be considered to be main outcome measures in the evaluation of Adriamycin-induced cardiotoxicity. It was also demonstrated that melatonin is significant for protecting against oxidation and lipid peroxidation, which is consistent with the results of previous studies. This may be one of the mechanisms by which melatonin reduced Adriamycin-induced myocardial injury. This is also consistent with the organ protective mechanism of melatonin through antioxidation observed in previous studies (21,21,25,31,32).

In the current study, it was observed that all rats treated with melatonin had a better overall quality of life. The tumors shrank in the ADT group, but the rats in this group had the worst quality of life and the shortest survival period (Table II). The tumors shrank slightly in the M+A group compared with that in the ADM group for application of melatonin, but the quality of life and survival period of the rats in the M+A group were improved compared with those in the ADM group. The treatment concept for advanced malignant tumors has changed. Compared with the previous focus simply on tumor shrinkage, at present, improvements to the quality of life and extension of the survival period are considered to be more important. It has been demonstrated in previous studies that melatonin relieves toxicity and enhances the curative effect in addition to improving the quality of life in the treatment of a variety of tumors (33–35), which was confirmed again by the current study, indicating that as an adjuvant drug of Adriamycin or other chemotherapy drugs, melatonin may have a function concordant with the current concept of tumor therapy. However, the mechanism of melatonin is very complicated. Further studies into possible mechanisms are required in order to make developments that are of clinical value.

## Figures and Tables

**Table I:** Comparison of LPO, SOD and GSH-Px concentrations in the various groups.

Groups	LPO (nmol/mgPr)	SOD (*μ*g/mgPr)	GSH-Px (*μ*/mgPr)
Blank	6.68±0.91	26.67±2.13	17.33±2.17
Diss	7.15±0.44	25.33±4.49	16.54±1.91
MLT	5.59±1.77	26.55±2.07	18.02±0.75
ADM	9.88±1.50	10.19±0.78	8.97±0.57
M+A	6.79±0.48^a^	19.99±1.91^b^	13.81±1.52^c^

a t=5.557, P= 0.025 vs. ADM group;

b t=−13.99, P=0.029 vs. ADM group;

c t=−8.462, P=0.014 vs. ADM group. Blank, no intervention; Diss, solvent intervention; MLT, melatonin intervention; ADM, Adriamycin intervention; M+A, melatonin + Adriamycin intervention; LPO, lipid peroxide; SOD, superoxide dismutase; GSH-Px, glutathione peroxidase.

**Table II:** Comparison of 1-month survival rate among groups.

Groups	n	One month survival (n)
Blank	16	4
Diss	16	6
MLT	14	14
ADM	16	5
M+A	14	11

Blank and Diss: χ^2^=1.45, P=0.35; Blank and MLT: χ^2^=17.5, P=0.000; Blank and ADM: χ^2^=0.00, P=1.00; Blank and M+A: χ^2^=8.57, P=0.005; MLT and M+A: χ^2^=3.36, P=0.1; ADM and M+A: χ^2^=6.71, P=0.012. Blank, no intervention; Diss, solvent intervention; MLT, melatonin intervention; ADM, Adriamycin intervention; M+A, melatonin + Adriamycin intervention.

## References

[1] Rock E, DeMichele A (2003). Nutritional approaches to late toxicities of adjuvant chemotherapy in breast cancer survivors. J Nutr.

[2] Billingham ME, Mason JW, Bristow MR, Daniels JR (1978). Anthracycline cardiomyopathy monitored by morphologic changes. Cancer Treat Rep.

[3] Bristow MR, Thompson PD, Martin RP, Mason JW, Billingham ME, Harrison DC (1978). Early anthracycline cardiotoxicity. Am J Med.

[4] Chicco AJ, Schneider CM, Hayward R (2005). Voluntary exercise protects against acute doxorubicin cardiotoxicity in the isolated perfused rat heart. Am J Physiol Regul Integr Comp Physiol.

[5] Chicco AJ, Schneider CM, Hayward R (2006). Exercise training attenuates acute doxorubicin-induced cardiac dysfunction. J Cardiovasc Pharmacol.

[6] Hydock DS, Lien CY, Schneider CM, Hayward R (2008). Exercise preconditioning protects against doxorubicin-induced cardiac dysfunction. Med Sci Sports Exerc.

[7] Albini A, Pennesi G, Donatelli F, Cammarota R, De Flora S, Noonan DM (2010). Cardiotoxicity of anticancer drugs: the need for cardio-oncology and cardio-oncological prevention. J Natl Cancer Inst.

[8] Lissoni P (2007). Biochemotherapy with immunomodulating pineal hormones other than melatonin: 5-methoxytryptamine as a new oncostatic pineal agent. Pathol Biol (Paris).

[9] Jawed S, Kim B, Ottenhof T, Brown GM, Werstiuk ES, Niles LP (2007). Human melatonin MT1 receptor induction by valproic acid and its effects in combination with melatonin on MCF-7 breast cancer cell proliferation. Eur J Pharmacol.

[10] Lemus-Wilson A, Kelly PA, Blask DE (1995). Melatonin blocks the stimulation effects of prolactin and human breast cancer cell growth in culture. Br J Cancer.

[11] Sánchez-Barceló EJ, Cos S, Mediavilla D, Martínez-Campa C, González A, Alonso-González C (2005). Melatonin-estrogen interactions in breast cancer. J Pineal Res.

[12] Zhang Y, Zhu S, Liu J (2009). The reverse effect and mechanism of melatonin on breast carcinoma cell line MCF-7/ADM resistant to Adriamycin. Chinese Journal of Clinical Oncology.

[13] Zhang Y, Zhu S, Zhao W (2009). The effect of melatonin on the sensitivity of ER^+^breast carcinoma cell line MCF-7 to Adriamycin and its mechanism. Chinese Journal of Clinical Oncology.

[14] Reiter RJ, Tan DX, Manchester LC, Paredes SD, Mayo JC, Sainz RM (2009). Melatonin and reproduction revisited. Biol Reprod.

[15] Sener G, Jahovic N, Tosun O, Atasoy BM, Yeğen BC (2003). Melatonin ameliorates ionizing radiation-induced oxidative organ damage in rats. Life Sci.

[16] Reiter RJ, Guerrero JM, Escames G, Pappolla MA, Acuña-Castroviejo D (1997). Prophylactic actions of melatonin in oxidative neurotoxicity. Ann N Y Acad Sci.

[17] Okatani Y, Wakatsuki A, Kaneda C (2000). Melatonin increases activities of glutathione peroxidase and superoxide dismutase in fetal rat brain. J Pineal Res.

[18] Reiter RJ, Acuña-Castroviejo D, Tan DX, Burkhardt S (2001). Free radical-mediated molecular damage. Mechanisms for the protective actions of melatonin in the central nervous system. Ann N Y Acad Sci.

[19] Hardeland R, Pandi-Perumal SR, Cardinali DP (2006). Melatonin. Int J Biochem Cell Biol.

[20] Ganguly K, Kundu P, Banerjee A, Reiter RJ, Swarnakar S (2006). Hydrogen peroxide-mediated downregulation of matrix metalloprotease-2 in indomethacin-induced acute gastric ulceration is blocked by melatonin and other antioxidants. Free Radic Biol Med.

[21] Bruck R, Aeed H, Avni Y (2004). Melatonin inhibits nuclear factor kappa B activation and oxidative stress and protects against thioacetamide induced liver damage in rats. J Hepatol.

[22] Yurtcu E, Guney Y, Ergun MA (2007). Lack of a time-dependent effect of melatonin on radiation-induced apoptosis in cultured rat lymphocytes. Cell Biol Int.

[23] Russo J, Tay LK, Russo IH (1982). Differentiation of the mammary gland and susceptibility to carcinogenesis. Breast Cancer Res Treat.

[24] Dagar S, Sekosan M, Rubinstein I, Onyüksel H (2001). Detection of VIP receptors in MNU-induced breast cancer in rats: implications for breast cancer targeting. Breast Cancer Res Treat.

[25] Sener-Muratoğlu G, Paskaloğlu K, Arbak S, Hürdağ C, Ayanoğlu-Dülger G (2001). Protective effect of famotidine, omeprazole, and melatonin against acetylsalicylic acid-induced gastric damage in rats. Dig Dis Sci.

[26] Maestroni GJ (2001). The immunotherapeutic potential of melatonin. Expert Opin Investig Drugs.

[27] Wang WZ, Fang XH, Stephenson LL (2005). Microcirculatory effects of melatonin in rat skeletal muscle after prolonged ischemia. J Pineal Res.

[28] Topal T, Oter S, Korkmaz A (2004). Exogenously administered and endogenously produced melatonin reduce hyperbaric oxygen-induced oxidative stress in rat lung. Life Sci.

[29] Fisher PW, Salloum F, Das A (2005). Phosphodiesterase-5 inhibition with sildenafil attenuates cardiomyocyte apoptosis and left ventricular dysfunction in a chronic model of doxorubicin cardiotoxicity. Circulation.

[30] Floyd JD, Nguyen DT, Lobins RL (2005). Cardiotoxicity of cancer therapy. J Clin Oncol.

[31] Mirunalini S, Karthishwaran K, Dhamodharan G, Shalini M (2010). Melatonin attenuates lipid peroxidation and enhances circulatory antioxidants during mammary carcinogenesis in rats. J Biochem Tech.

[32] Reiter RJ, Tan DX (2003). Melatonin: a novel protective agent against oxidative injury of the ischemic/reperfused heart. Cardiovasc Res.

[33] Lissoni P, Barni S, Mandalá M, Ardizzoia A, Paolorossi F (1999). Decreased toxicity and increased efficacy of cancer chemotherapy using the pineal hormone melatonin in metastatic solid tumour patients with poor clinical status. Eur J Cancer.

[34] Sánchez-Suárez P, Ostrosky-Wegman P, Gallegos-Hernández F (2008). DNA damage in peripheral blood lymphocytes in patients during combined chemotherapy for breast cancer. Mutat Res.

[35] Lissoni P (2007). Biochemotherapy with standard chemotherapies plus the pineal hormone melatonin in the treatment of advanced solid neoplasms. Pathol Biol (Paris).

